# Upcycling cheese whey permeate into fully bio-based surfactants through fermentation and biocatalysis

**DOI:** 10.1007/s00253-025-13630-y

**Published:** 2025-12-18

**Authors:** Riccardo Semproli, Lorenza Cassano, Giorgia Ballabio, Giuseppe Cappelletti, Giovanna Speranza, Silvia Donzella, Concetta Compagno, Daniela Ubiali, Marina Simona Robescu

**Affiliations:** 1 Department of Drug Sciences, University of Pavia, Viale Taramelli 12, 27100 Pavia, Italy; 2 Department of Chemistry, University of Milan, Via Golgi 19, 20133 Milan, Italy; 3 Department of Food, Nutrition and Environmental Sciences, University of Milan, Via Mangiagalli 25, 20133 Milan, Italy

**Keywords:** Cheese whey permeate, Biomass upcycling, Biocatalysis, Fermentation, Sugar-based surfactant

## Abstract

**Abstract:**

Whey permeate (WP), the main waste stream of the dairy industry, was used as a raw material to produce fully bio-based non-ionic surfactants. Specifically, on the one hand, WP was submitted to a transglycosylation reaction catalyzed by the immobilized β-galactosidase from *Aspergillus oryzae* in 1-BuOH, affording 1-butyl β-D-galactopyranoside (yield 40%), which was used as the polar “head” of the surfactant. On the other hand, a WP-based fermentation process by the yeast *Cutaneotrichosporon oleaginosus* ATCC 20509 was employed to produce single cell oil (45% w/w_cell dry weight_). The microbial lipids were recovered from the freeze-dried cells and derivatized in a one-pot system by acid-catalysis to yield the corresponding ethyl esters as apolar “tails” (75% w/w yield, based on lipid content). These bio-based building blocks were converted into the sugar fatty acid esters (SFAE) *n*-butyl 6-*O*-acyl-β-D-galactopyranosides by a lipase-catalyzed transesterification reaction (yield 40%). The hydrophilic–lipophilic balance and solubility parameters of the synthesized SFAE mixture were calculated. Additionally, its ability to reduce the interfacial tension was examined, including the effect of fatty tail unsaturation. The interfacial performance of the SFAE mixture, containing both palmitic (45%) and oleic (40%) acid residues, was lower (6.3 mN m⁻^1^) compared to the SFAE containing only palmitic acid as the fatty acid tail (0.1 mN m⁻^1^) at the same concentration, but still highly promising.

**Key points:**

• *Whey permeate (WP) is the main waste stream of dairy industry.*

• *WP was upcycled by coupling fermentation and biocatalysis.*

• *Bio-based surfactants (sugar fatty acid esters) were obtained using WP as biomass.*

**Graphical Abstract:**

**Figure:**
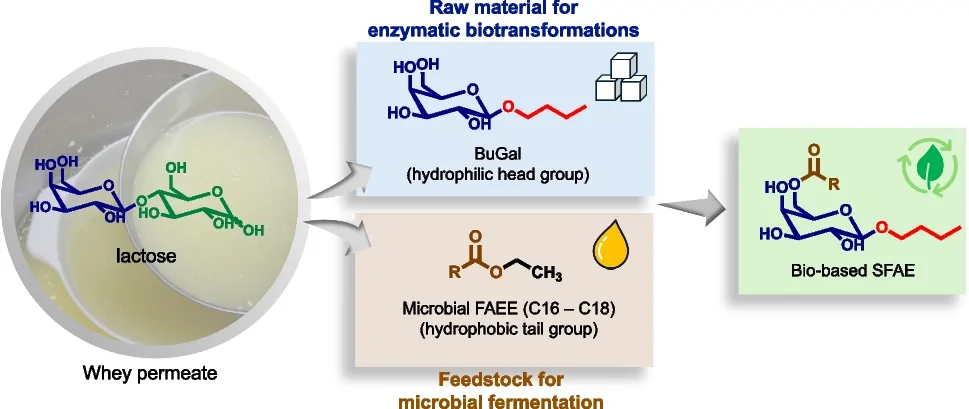

**Supplementary Information:**

The online version contains supplementary material available at 10.1007/s00253-025-13630-y.

## Introduction

The consumption of dairy products is a widespread and well-established dietary practice across the globe, with over 80% of the population consuming them regularly (Food and Agriculture Organization of the United Nations (FAO) 2019). In 2024, worldwide milk production reached 982 million tons, with 236 million tons produced in Europe alone (Food and Agriculture Organization of the United Nations (FAO) 2024). The cheesemaking process produces large volumes of wastewaters: for every 100 kg of milk, only 10 kg of cheese are produced, resulting in 90 kg of whey as the main by-product (Usmani et al. 2022). Whey is a complex mixture of proteins (6 − 8 g L⁻^1^), a significant amount of lactose (45 − 50 g L⁻^1^), as well as mineral salts (8 − 10%), fats (4 − 5 g L⁻^1^), and B vitamins (Zotta et al. 2020). These constituents lead to high levels of chemical oxygen demand (COD) (80–95 g/L) and biological oxygen demand (BOD) (40–48 g/L), making proper treatment essential before disposal (Ahmad et al. 2019).

Proteins are usually recovered from whey through ultrafiltration and used for food and nutraceutical products (Pires et al. 2021). The residue from ultrafiltration is whey permeate (WP), which contains mainly lactose, the main contributor to the high COD values (> 70%) (Ahmad et al. 2019; Pires et al. 2021).

Following the principles of the circular economy framework, WP can be transformed from a highly polluting by-product into a low-cost renewable biomass, non-seasonal, independent of climate, and not in competition with the food supply chains to produce new products (Homrich et al. 2018). WP was exploited as a carbon source to support the growth of microbial cell factories for the manufacturing of products with high industrial interest (Goyal et al. 2023) such as carboxylic acids (acetic, propionic, lactic, and succinic acids) (Pais-Chanfrau et al. 2020), biofuels (bioethanol, biobutanol, biogas) (Pasotti et al. 2017; Osorio-González et al. 2022; Ma et al. 2024), biopolymers (Zikmanis et al. 2020; Wang et al. 2025), antioxidants (Costa et al. 2022), and single cell oil (SCO) (Chan et al. 2018; Donzella et al. 2022; Kiani et al. 2024). WP was also successfully used as a low-cost inducer in the recombinant production of different classes of enzymes of industrial interest (Greicius et al. 2023; Bianchi et al. 2024). Eventually, lactose contained in WP can be enzymatically or chemically transformed into high-added value products such as prebiotic carbohydrates (galacto-oligosaccharides, lactulose) (Fischer and Kleinschmidt 2018; Enteshari and Martínez-Monteagudo 2020), low-calorie sweetener (d-tagatose) (Wanarska and Kur 2012; Jayamuthunagai et al. 2017; Cervantes et al. 2020; Zhang et al. 2022), and sugar-based surfactants (glucose/galactose fatty acid esters) (Ballabio et al. 2024).

Sugar fatty acid esters (SFAE) are non-ionic surfactants having a sugar moiety as a polar “head” and a fatty acid “tail.” SFAE show excellent emulsifying, stabilizing, and detergency properties. Furthermore, they are appealing for many applications since they are tasteless, odorless, nontoxic, non-harmful to the environment, and fully biodegradable (Perez et al. 2017, Verboni et al. 2023). Fully bio-based SFAE can be easily obtained since their building blocks can be bio-derived from abundant, renewable resources such as sugars and oils or derived from waste upcycling (Pyo et al. 2019).

The use of renewable feedstocks in chemical manufacturing can reduce the environmental impact and production costs. Additionally, coupling renewable feedstocks with sustainable synthetic schemes is crucial for creating more efficient and environmentally friendly processes. This combination would minimize waste and energy use, also aligning with end-user expectations. The chemical synthesis of SFAE generally requires harsh reaction conditions (high temperature, acid/base catalysts, and hazardous solvents), resulting in intensive energy consumption, formation of undesired by-products, and/or heterogeneous mixtures of variably esterified and acylated products (Pyo et al. 2019).

Lipases can be used as biocatalysts for coupling the sugar moiety with the apolar tail in milder and more environmentally friendly conditions. Owing to the regioselectivity of lipases, a narrow product distribution is generally achieved (Pyo et al. 2019).

**Fig. 1:**
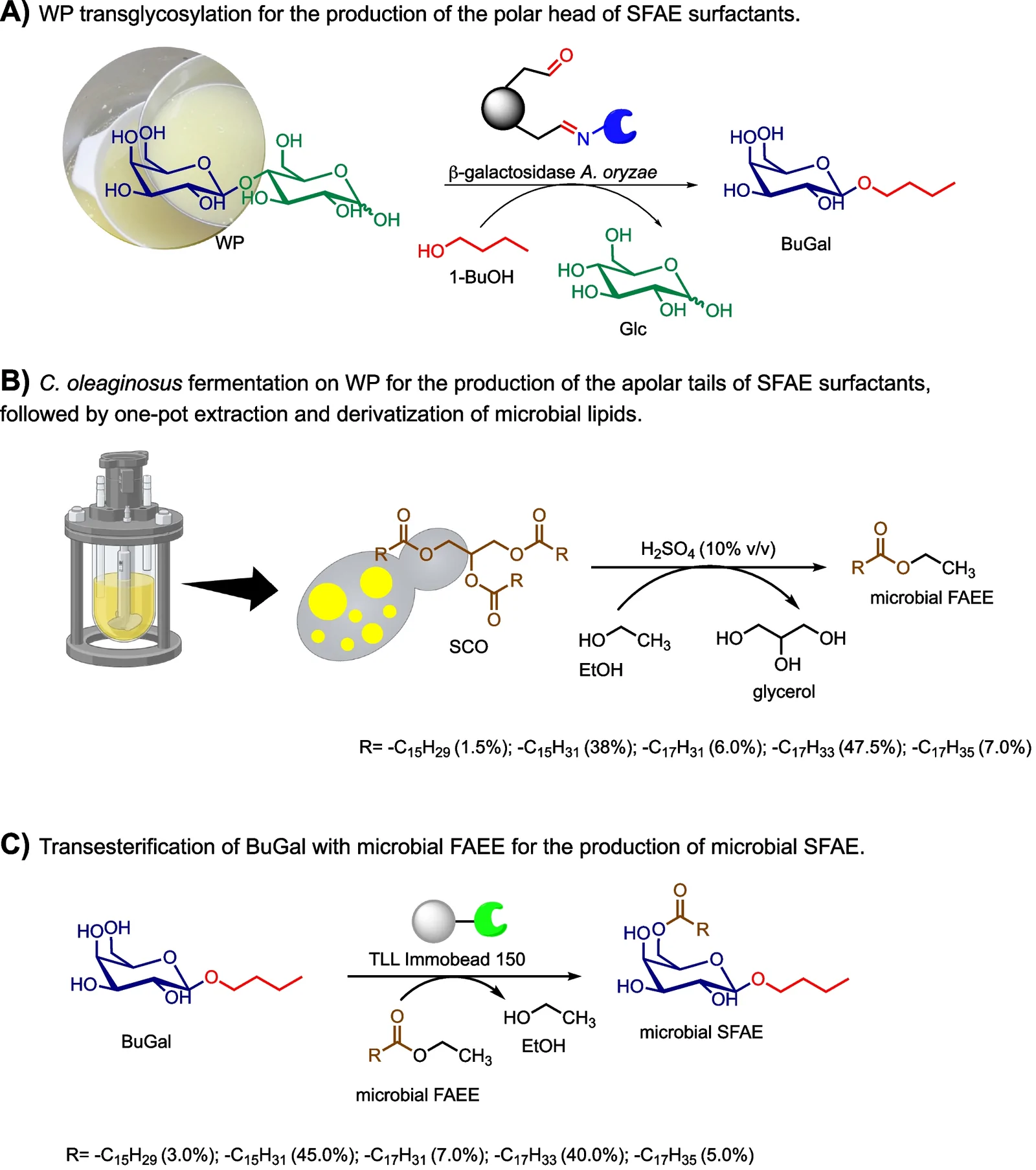
Workflow towards fully bio-based SFAE from WP: **A** Enzymatic transglycosylation of WP in 1-BuOH catalyzed by immobilized β-galactosidase from *Aspergillus oryzae*. **B** Production, one-pot extraction, and derivatization of microbial lipids into microbial fatty acid ethyl esters (FAEE). **C** Solvent-free enzymatic synthesis of sugar fatty acid esters (SFAE) starting from the bio-based building blocks obtained in step A and step B

Here, we describe an integrated platform for the complete upcycling of cheese whey permeate into fully bio-based sugar ester surfactants through biocatalysis and fermentation. On the one hand, lactose contained in WP was enzymatically transformed into the galactose-based polar head of the surfactants. On the other hand, WP was the feedstock for the fermentation process by an oleaginous yeast to produce SCO as the apolar tails of the surfactants. In this system, liquid WP was exploited not only as a source of substrates but also as a source of water to reduce the water footprint of both biotransformation and fermentation processes. These bio-based intermediates, once produced, were reacted by a lipase-mediated transesterification to obtain the desired sugar ester surfactants (Fig. 1).

## Materials and methods

### General

WP was kindly provided by Latteria Soresina (Italy). Microbial lipids were produced through a WP-based fermentation process by the yeast *Cutaneotrichosporon oleaginosus* ATCC 20509*,* as previously reported (Donzella et al. 2022).

Sepabeads^® ^EC-EP/S was kindly supplied by Resindion (Italy), activated to glyoxyl Sepabeads, and then used for the immobilization of β-galactosidase from *Aspergillus oryzae* (Gal*Ao*), as previously reported (Semproli et al. 2023). Immobilized lipase B from *Candida antarctica* (Novozym^®^ 435) was kindly supplied by Novozymes (Denmark). TLL Immobead 150 was supplied by Merck Life Science s.r.l. (Italy). All reagents and solvents were purchased from Merck Life Science s.r.l. (Italy) if not otherwise stated.

HPLC analyses were performed using an HPLC Chromaster (VWR Hitachi Chromaster, Japan) equipped with a 5310 column oven, a 5260 auto sampler, a 5160 pump, and an ELSD detector (SEDEX 100LT). 1-Butyl β-d-galactopyranoside (BuGal) used for the HPLC calibration curve was obtained starting from commercial lactose, as previously reported (Semproli et al. 2023).

^1^H-NMR and ^13^C-NMR spectra were recorded at 400.13 MHz and 100 MHz, respectively, on a Bruker AVANCE 400 MHz equipped with TOPSPIN software (Bruker, Germany) at 300 K. Chemical shifts (δ) are expressed in ppm and referred to the solvent signals (DMSO-*d*_6_, δ_H_ 2.50 ppm and δ_C_ 39.52 ppm from TMS).

### Characterization of WP

The concentration of lactose and the traces of galactose contained in WP were determined by using the Lactose/d-Galactose Rapid Assay Kit (K-LACGAR) from Megazyme NEOGEN (Ireland) following supplier indications. Traces of d-glucose contained in WP were determined by using the GOPOD kit from Megazyme NEOGEN (Ireland) following supplier indications.

### Enzymatic transglycosylation of commercial lactose for the synthesis of 1-butyl β-d-galactopyranoside: small scale

Anhydrous lactose (90 mg, 0.26 mmol) was solubilized in McIlvaine buffer pH 4.5 (2 mL) in a glass vial. A ternary homogeneous system was generated by adding to this solution 1-BuOH (5 mL) and acetone (3 mL). The reaction was started by the addition of the immobilized GalAo (200 mg, 0.2 IU), and the mixture was incubated (orbital shaker) at 30 °C for 24 h. At different endpoints, samples were withdrawn (100 μL) from the reaction mixture, dried under N_2_ flow, then solubilized in H_2_O (100 μL), and analyzed by HPLC. Conversion was determined based on initial lactose content.

### Enzymatic transglycosylation of WP for the synthesis of 1-butyl β-d-galactopyranoside: small scale

WP (45 g L^−1^ lactose) was directly used in the transglycosylation reaction in 1-BuOH. The pH of WP was adjusted to 4.5 with HCl 1 M. WP (2 mL, corresponding to 0.26 mmol of lactose), 1-BuOH (5 mL), and acetone (3 mL) were mixed in a glass vial. The immobilized GalAo (200 mg, 0.2 IU) was added to the reaction, and the mixture was incubated (orbital shaker) at 30 °C for 24 h. At different endpoints, samples were withdrawn (100 μL) from the reaction mixture, dried under N_2_ flow, then solubilized in H_2_O (100 μL), and analyzed by HPLC. Conversion was determined based on initial lactose content.

### Enzymatic transglycosylation of WP for the synthesis of 1-butyl β-d-galactopyranoside: scale-up and purification

The pH of WP (45 g L^−1^) was adjusted to 4.5 with HCl 1 M. WP (20 mL, corresponding to 2.6 mmol of lactose), 1-BuOH (50 mL), and acetone (30 mL) were mixed in a round-bottom flask. The immobilized GalAo (2 g, 2 IU) was added to the reaction, and the mixture was maintained under magnetic stirring at 30 °C for 2.5 h. The reaction was stopped by filtration of the enzyme, and then the immobilized enzyme was washed with 1-BuOH (10 mL). The filtrate was evaporated under reduced pressure; the crude was solubilized in MeOH and added with silica (≈ 2.5 g) to adsorb the components of the reaction mixture. The suspension was dried under reduced pressure and dried silica was loaded on top of a packed silica column for flash chromatography purification (DCM/MeOH, 90:10). The purification was monitored by TLC (DCM/MeOH, 85:15) by using H_2_SO_4_ 5% v/v in EtOH and heating for detection (Rf_BuGal_ = 0.25, Rf_Glc/Gal_ = 0.05, Rf_Lac_ = 0). The product, obtained as a light-yellow powder (yield: 40%), was characterized by ^1^H-NMR analysis (Supplementary Fig. 1).

### HPLC-ELSD monitoring of the transglycosylation reaction

HPLC analyses were carried out by using a Supelco C-610H column (30 cm × 7.8 mm) (59320-U, Sigma-Aldrich) and 0.1% formic acid in water as the mobile phase. The column was thermostated at 30 °C, the flow rate was set at 0.5 mL/min, the injection volume was 10 μL, and the analysis time was 23 min. The detection was carried out by using an evaporative light scattering detector applying the following parameters: temperature: 60 °C; gain: dynamic; filter: 10 s; P: 3.5 bar. Retention times: lactose (Lac) = 10.9 min; glucose (Glc) = 12.5 min; galactose (Gal) = 13.4 min; BuGal = 19.0 min.

### Solvent-free enzymatic SFAE synthesis optimization (general protocol)

BuGal (150 mg; 0.64 mmol), the proper acyl donor (palmitic acid: PA, palmitic acid methyl or ethyl ester: PAME or PAEE, respectively; 0.64–5.25 mmol), activated 4 Å molecular sieves, and immobilized lipase (Novozym 435 or TLL Immobead 150, 10% w/w) were poured into a round-bottom flask and incubated at 80 °C (oil bath) under magnetic stirring (900 rpm) for 8 h. The flask was connected to a vacuum pump, which was periodically switched on to remove the water/alcohol released during the reaction. At the endpoint, the mixture was diluted with ethyl acetate (5–20 mL), the enzyme was eliminated by decantation, and the solvent was evaporated under reduced pressure. The crude was solubilized in DCM/MeOH 80:20 and added to silica (≈ 2.5 g) to adsorb the components of the reaction mixture. The suspension was dried under reduced pressure, and the dried silica was loaded on top of a packed silica column for flash chromatography purification (EtOAc/*n*-Hex, gradient: from 50:50 to 80:20). The purification was monitored by TLC (EtOAc/*n*-Hex, 80:20) using Ce(SO_4_)_2_/(NH_4_)_6_Mo_7_O_24_ × 4H_2_O in 6% v/v sulfuric acid in H_2_O and heating for detection. The pure SFAE were obtained with a variable yield depending on the acyl donor (5–50% yield).

### One-pot extraction and derivatization of microbial lipids by acid-catalyzed in-situ transesterification

*C. oleaginosus* ATCC 20509 freeze-dried cells (10 g, containing 45% w/w microbial lipids, corresponding to 4.5 g) were suspended in 10% v/v H_2_SO_4_ in anhydrous EtOH (110 mL) at 70 °C under magnetic stirring for 8 h in the presence of 4 Å molecular sieves. The downstream was performed as reported in the literature with slight modifications (Bavaro et al. 2020). At the endpoint, 10% w/v aqueous NaCl (100 mL) was added, and the mixture was extracted with *n-*heptane (6 × 100 mL). The organic phases were collected, dried over anhydrous Na_2_SO_4_, and the solvent was removed under reduced pressure, thus obtaining crude microbial fatty acid ethyl esters (FAEE) containing free fatty acids (FFA) as impurities. The crude was dissolved in *n-*hexane and incubated with Poligoprep^®^ 60–30 NH_2_ resin (50% w/w) under magnetic stirring for 10 min to remove FFA. Then, the resin was filtered off, and the solvent was removed under reduced pressure. Pure microbial FAEE were obtained as an orange oil (3.37 g; 75% w/w yield based on lipid content). The fatty acid composition of the obtained FAEE was determined by GC–MS as previously reported (Donzella et al. 2022) (Supplementary Fig. 2A).

### Solvent-free enzymatic synthesis of microbial SFAE by transesterification

BuGal (300 mg; 1.27 mmol), microbial FAEE (1.18 g; 3.8 mmol), and TLL Immobead 150 (10% w/w) were poured into a 5-mL round bottom flask and incubated at 80 °C (oil bath) under magnetic stirring (900 rpm) for 8 h. The flask was connected to a vacuum pump, which was periodically switched on to remove EtOH released during the reaction. At the endpoint, the mixture was diluted with ethyl acetate (10 mL), the enzyme was eliminated by decantation, and the solvent was evaporated under reduced pressure. The crude was solubilized in DCM/MeOH 80:20 and added to silica (≈ 2.5 g) to adsorb the components of the reaction mixture. The suspension was dried under reduced pressure, and the dried silica was loaded on top of a packed silica column for flash chromatography purification (EtOAc/*n*-Hex, gradient: from 50:50 to 80:20). The purification was monitored by TLC (EtOAc/*n*-Hex, 80:20) using Ce(SO_4_)_2_/(NH_4_)_6_Mo_7_O_24_ × 4H_2_O in 6% v/v sulfuric acid in H_2_O and heating for detection (Rf_BuGal_ = 0, Rf_microbial SFAE_ = 0.35, Rf_FFA_ = 0.85, Rf_microbial FAEE_ = 1). The obtained mixture of SFAE (40% yield) was characterized by GC–MS (Supplementary Fig. 2B), ^1^H-NMR, and ^13^C-NMR (Supplementary Fig. 3). For GC–MS analyses, microbial SFAE were converted into the corresponding FAME by base-catalyzed methanolysis. Briefly, microbial SFAE (10 mg) were dissolved in *n-*heptane (0.5 mL), and a 10% (w/v) KOH solution in MeOH (0.020 mL) was added. The mixture was incubated at 37 °C for 10 min and then quenched by the addition of KHSO_4_ (50 mg). The suspension was centrifuged, and the supernatant was diluted (10×) with *n-*heptane for the analysis to determine the composition of hydrophobic tails. The fatty acid composition was analyzed as previously reported (Donzella et al. 2022).

### Solubility calculation

Hydrophilic–lipophilic balance (HLB) values were calculated according to the Griffin method (Griffin 1949):

1 $$HLB=20*\frac{{M}_{H}}{M}$$

where ${M}_{H}$ is the molecular mass of the hydrophilic portion of the molecule whereas M is the molecular mass of the whole molecule.

To predict the solubility of the synthesized surfactant, the δ_D_, δ_P_, δ_H_, and δ_T_ parameters for palmitoyl (BuGal C16), palmitoleyl (BuGal C16:1), linoleyl (BuGal C18:2), oleyl (BuGal C18:1), and stearyl (BuGal C18) derivatives were calculated using the Hansen solubility model. These values were used to determine the relative weighted *δ* values for *n*-butyl 6-*O*-acyl galactopyranoside mixture. The incremental method (Eqs. 2–5) was used to estimate the solubility considering the contribution of the different groups.

2 $$\delta_D=\frac{\sum F_{Di}}{\sum V_i}$$

3 $$\delta_P=\frac{\sqrt{\sum F_{Pi}^2}}{\sum V_i}$$

4 $$\delta_H=\sqrt{\frac{\sum E_{Hi}}{\sum V_i}}$$

5 $$\delta_T^2=\delta_D^2+\delta_P^2+\delta_H^2$$

where δ_T_, δ_D_, δ_P_, and δ_H_ are the total, dispersive, polar, and hydrogen bonding partial solubility parameters, respectively. *V* is the group contribution to molar volume, and *F*_Di_, *F*_Pi_, and *E*_Hi_ are the group attraction constants for dispersion (*F*_Di_), polar components (*F*_Pi_), and group cohesion energies (*E*_Hi_) (Xavier-Junior et al. 2016).

The group increments for the calculation of the Hansen solubility parameters were taken from literature (Mollet and Grubenmann 2001). The solubility differences (Δδ) between surfactants and solvents (i.e., water and sunflower oil) were also calculated.

### Interfacial tension (IFT) measurements

Interfacial tension (IFT) at the sunflower oil/water interface was measured for *n*-butyl 6-*O*-acyl-galactopyranoside mixture at concentrations of 0.04, 0.7, and 1.4 g L⁻^1^. Measurements were conducted at (25 ± 1) °C using a Gibertini tensiometer (Du Noüy ring method), applying Harkins–Jordan corrections after 1 h of equilibration. The resulting IFT values were compared to previously reported data for *n*-butyl 6-*O*-palmitoyl-β-d-galactopyranoside (Semproli et al. 2023). Additionally, *n*-butyl 6-*O*-oleoyl-β-d-galactopyranoside was synthesized as a reference standard (unpublished results) (Supplementary Fig. 4), and its IFT values were determined under the same conditions. All values are reported as averages from three replicate measurements.

## Results

### Synthesis of 1-butyl β-d-galactopyranoside by enzymatic transglycosylation of WP

The synthesis of SFAE is difficult due to the remarkable polarity difference between the sugar head and the fatty acid tail (Pyo et al. 2019). One well-established strategy for the synthesis of SFAE requires the conversion of the sugar moiety into a less polar derivative, followed by solvent-free esterification (Sangiorgio et al. 2022, 2024; Semproli et al. 2023; Ballabio et al. 2024).

In a previous work, SFAE were successfully synthesized by a two-step enzymatic process starting from commercial substrates (Semproli et al. 2023). First, the galactose-based polar head of SFAE was obtained by transforming lactose (10 mM) into 1-butyl β-d-galactopyranoside (BuGal) (yield: 45%) through a transglycosylation reaction catalyzed by an immobilized β-galactosidase from *Aspergillus oryzae* (GalAo) in a homogeneous ternary system (20/30/50 aqueous phase/acetone/1-BuOH). This building block was esterified with commercial palmitic acid in a solvent-free system, yielding the corresponding SFAE (15% yield), which showed effective emulsifying properties.

Starting from these results, in this paper we evaluated the use of WP, instead of commercial lactose, to obtain fully bio-based surfactants by applying the same biotransformation to the biomass (Fig. 1A).

The sugar content of WP was assessed by commercial enzymatic kits. WP used in this work contained 45 g L^−1^ lactose with traces of glucose (0.09 g L^−1^) and galactose (0.03 g L^−1^). To completely exploit the potential of liquid WP, we investigated its use as a dual-purpose source: WP can provide, indeed, both the substrate (lactose) and the water required for the biotransformation, thus minimizing the overall water footprint. The reaction was set up as previously reported (Semproli et al. 2023), by replacing completely the aqueous phase (McIlvaine buffer pH 4.5) with WP (45 g L^−1^ lactose, 26 mM, pH 6.7). Since the optimum pH required for the biocatalyst activity is reported around pH 4.5 (Maksimainen et al. 2013), we adjusted its pH to this value.

**Fig. 2:**
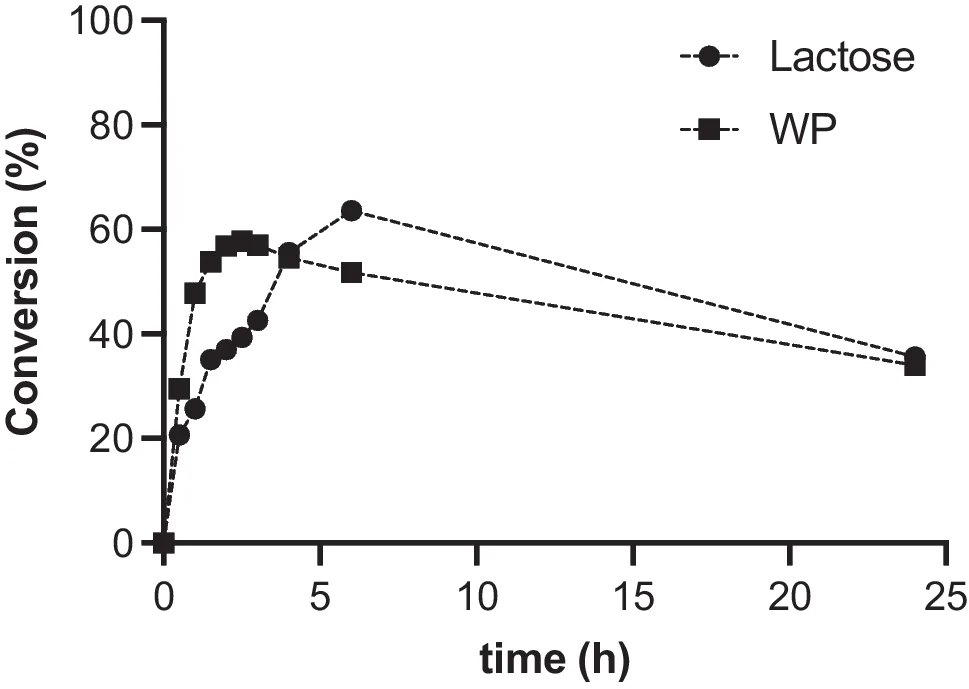
Time course of BuGal formation by enzymatic transglycosylation from commercial lactose (circles) and WP (squares). Experimental conditions: 0.26 mmol lactose in McIlvaine buffer pH 4.5 (2 mL) or WP (45 g L^-1^ lactose; 2 mL; 0.26 mmol lactose) pH 4.5, acetone (3 mL), 1-BuOH (5 mL), immobilized GalAo (200 mg, 0.2 UI), 30 °C, 24 h. Conversion was calculated based on initial lactose content

The use of WP without any dilution resulted in a 2.5 × increase in lactose concentration compared to the standard reaction. To evaluate the impact of a higher concentration of starting lactose, we set up two small-scale biotransformations by using both commercial lactose and WP, and we monitored these reactions by HPLC-ELSD for 24 h (Supplementary Fig. 5). As shown in Fig. 2, both biotransformations reached the same maximum conversion (~ 60%), but the use of WP led to a shorter reaction time (2.5 h instead of 6 h). A decrease in BuGal concentration was observed upon extended incubation time for both reactions (Fig. 2) due to the hydrolytic activity of the enzyme that eventually starts to prevail over the transglycosylation activity. At this point, the reaction with WP was scaled up (10×), and the product (BuGal) was isolated in good yield (40%). The product was characterized by ^1^H-NMR (Supplementary Fig. 1), and its chemical purity was determined by HPLC-ELSD.

### Synthesis of *n*-butyl 6-*O*-palmitoyl galactopyranoside by enzymatic solvent-free (trans)esterification: Reaction conditions optimization

**Fig. 3:**
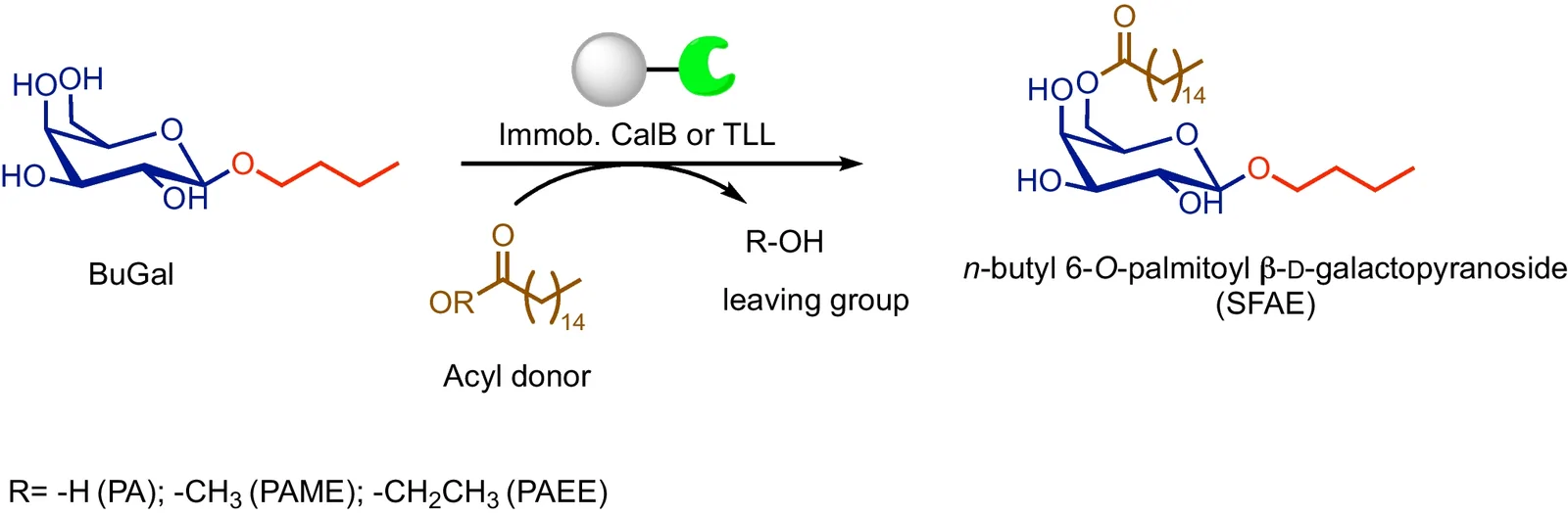
Synthesis of SFAE (*n*-butyl 6-*O*-palmitoyl-β-d-galactopyranoside) by enzymatic (trans)esterification catalyzed by immobilized lipases. PA: palmitic acid, PAME: palmitic acid methyl ester, PAEE: palmitic acid ethyl ester

At this point, we focused our attention on the solvent-free acylation step by using bio-based BuGal as the substrate (Fig. 3).

In a previous work (Semproli et al. 2023), this step was performed by direct esterification with PA catalyzed by CalB in a glass oven B-585 Kugelrohr and resulted in a 15% yield (Table 1,entry 1). As a starting point for the SFAE synthesis optimization, these conditions were tested (except for the stirring system) using both CalB and TLL. The latter was included in this study because TLL was extensively reported to catalyze the synthesis of lactose fatty acid esters (Walsh et al. 2009; Liang et al. 2018) with a complete regioselectivity for the primary group of the galactose residue (C-6). Furthermore, a qualitative comparison (TLC) of the direct esterification reaction indicated that the reaction was cleaner when catalyzed by TLL than by CalB (Supplementary Fig. 6). However, < 10% yields were obtained with both enzymes (Table 1,entry 2–3). The yield increased only when using a large excess of PA (Table 1, entry 4), which negatively affected both product downstream and overall process economics. The main drawback of direct esterification is the release of water, which can cause the hydrolysis of the resulting SFAE. Water removal is particularly challenging in carbohydrate esterification compared to other esterification reactions due to the strong binding between saccharides and water molecules. In the transesterification reaction with PAME or PAEE, the formation of MeOH or EtOH as co-products allows for their easy removal, which drives the reaction equilibrium towards SFAE formation. Additionally, in solvent-free systems that use a stoichiometric excess of long-chain fatty acids or esters (which may be viscous liquid or even solids at room temperature), the viscosity of the medium becomes a critical factor impacting mass transfer and should therefore be considered (Sousa et al. 2021). In transesterification reactions, using esters as acyl donors with a melting point lower than that of the corresponding free fatty acid (PAME m.p. 35 °C, PAEE m.p. 26 °C, and PA m.p. 63 °C) is expected to decrease the viscosity of the reaction medium. This, in turn, helps to reduce potential mass transfer limitations compared to the esterification reaction.

**Table 1:** Synthesis of *n*-butyl 6-*O*-palmitoyl-β-d-galactopyranoside by enzymatic (trans)esterification

Entry	Donor	Ratio BuGal:donor	Enzyme	Yield (%)
1	PA	1:1	CALB	15^a^
2	PA	1:1	CALB	7
3	PA	1:1	TLL	5
4	PA	1:25	TLL	20
5	PAME	1:3	TLL	35
6	PAEE	1:3	TLL	50
7	PAEE	1:8	TLL	45

All the reactions were performed at 80 °C, 900 rpm using 10% w/w immobilized enzyme (referred to as the limiting reagent BuGal). *PA* palmitic acid, *PAME* palmitic acid methyl ester, *PAEE* palmitic acid ethyl ester ^a^As reported in Semproli et al. (2023)

As expected, the transesterification approach gave higher yields by using a lower excess of acyl donor. A slightly lower yield (35%, Table 1, entry 5) was obtained with FAME than with FAEE (50%, Table 1, entries 6–7). The inactivation of lipases by short-chain alcohols is a well-known and studied phenomenon in the biodiesel production process, with MeOH showing a stronger effect compared to EtOH.

The highest yield (50%) was obtained with TLL Immobead 150, PAEE, and a 1:3 ratio of reactants (BuGal: PAEE) (Table 1, entry 6). The NMR analyses confirmed the identity of *n*-butyl 6-*O*-palmitoyl-β-d-galactopyranoside.

### One-pot extraction and derivatization of microbial lipids by acid-catalyzed in-situ transesterification

Single cell oil (SCO) was obtained through a two-step fermentation process that employs the oleaginous yeast *Cutaneotrichosporon oleaginosus* ATCC20509. The cultivation in the bioreactor was carried out from WP supplemented with urea as a cheap nitrogen source, while candied mango syrup, another food industry by-product, served as a carbon feed. At the end of the fermentation, when the process achieved a 38 g L^−1^ productivity, the cells were recovered by centrifugation and freeze-dried. The lipid content reached 45% of cell dry weight, with the majority of lipids accumulated as triglycerides (77%) (Donzella et al. 2022).

The use of crude triglycerides as acyl donors for transesterification reactions is feasible, but it generally results in a complex reaction mixture containing, besides the desired products (SFAE), also diglycerides (DAG), monoglycerides (MAG), and free fatty acids (FFA) which are hard to remove. This was experimentally confirmed by incubating BuGal with a crude vegetable oil in the presence of TLL Immobead 150. A mixture of SFAE was obtained, but still containing traces of MAG, DAG, and FFA even after a chromatographic step (data not shown).

Based on the results we got with commercial PAEE, and the need to obtain microbial hydrophobic tails in a form suitable for the transesterification reaction, one-pot extraction and in situ derivatization of microbial lipids into fatty acid ethyl esters (microbial FAEE) were performed (Fig. 1B).

Freeze dried cells (10 g), containing about 4.5 g of SCO, were reacted through an in situ acid-catalyzed transesterification in EtOH, affording 3.37 g of microbial FAEE (75% w/w yield based on lipid content). The microbial FAEE were mainly composed of long-chain fatty acids (C16 and C18), consisting of saturated fatty acid chains (38% palmitic acid (C16) and 7% stearic acid (C18)) and unsaturated fatty acid chains (1.5% palmitoleic acid (C16:1), 6% linoleic acid (C18:2), and 47.5% oleic acid (C18:1)).

### Solvent-free enzymatic synthesis of fully bio-based SFAE

The final step of the integrated process developed in this work consisted of the incubation of the bio-based polar head (BuGal) with the bio-based hydrophobic tails (microbial FAEE) in the conditions set up by using commercial PAEE (ratio BuGal:microbial FAEE = 1:3, TLL Immobead 150 10% w/w, 80 °C, 8 h) (Fig. 1C). After chromatography, fully bio-based SFAE were obtained as variably acylated products depending on the microbial lipid composition: 3% palmitoleic acid (C16:1), 45% palmitic acid (C16), 7% linoleic acid (C18:2), 40% oleic acid (C18:1), and 5% stearic acid (C18) (Supplementary Fig. 2B). The reaction yield (40%) was consistent with the result obtained when using commercial PAEE. The SFAE mixture was characterized by ^1^H-NMR and ^13^C-NMR (Supplementary Fig. 3).

### Solubility calculation and interfacial tension (IFT) measurements

The hydrophilic-lipophilic balance (HLB) is a dimensionless value that indicates the affinity of a surfactant for either the oil or aqueous phase. Surfactants with HLB values between 0 and 9 are typically oil-soluble, while those with values between 11 and 20 show greater affinity for water (Mollet and Grubenmann 2001). Specifically, the synthesized “microbial” SFAE has an average weighted HLB value of 9.6, calculated using the Griffin method (Griffin 1949) (Table 2). This value lies near the boundary between oil and water solubility, indicating that the mixture may exhibit partial solubility in both phases. Thus, to gain deeper insight into the surfactants’ affinity for oil or water, Hansen solubility parameters were calculated. These parameters are widely used to predict intermolecular interactions, thereby aiding in the assessment of miscibility and solubility of chemical compounds (Xavier-Junior et al. 2016). Compounds with similar δ_T_ exhibit comparable intermolecular interaction forces, resulting in good solubility (Δδ < 7 MPa^1/2^) (Tsakiridou et al. 2019). The solubility of various *n*-butyl galactopyranoside derivatives—palmitoyl (BuGal C16), palmitoleyl (BuGal C16:1), linoleyl (BuGal C18:2), oleyl (BuGal C18:1), and stearyl (BuGal C18)—derivatives as well as the microbial SFAE mixture (as average weighted values), was calculated in water (δ_T_ = 47.8 MPa^1/2^) (Mollet and Grubenmann 2001) and in sunflower oil (δ_T_ = 16.8 MPa^1/2^) (Li et al. 2014). Based on the calculated δ_T_ values, both the individual SFAE and the mixture exhibit good solubility in sunflower oil, with Δδ < 4.5 MPa^1/2^ (Table 2).

**Table 2:** Hydrophilic-lipophilic balance (HLB), solubility parameters (δ_D_, δ_P_, δ_H_, and δ_T_), and the calculated Δδ values for palmitoyl (BuGal C16), palmitoleyl (BuGal C16:1), linoleyl (BuGal C18:2), oleyl (BuGal C18:1), and stearyl (BuGal C18) derivatives, along with the corresponding average weighted values for the microbial SFAE mixture

Compound	HLB	δ_D_/(MPa^1/2^)	δ_P_/(MPa^1/2^)	δ_H_/(MPa^1/2^)	δ_T_/(MPa^1/2^)	Δδ/(MPa^1/2^)	
						**Water**	**Sunflower oil**
BuGal C16	9.9	16.6	2.7	13.1	21.3	26.5	4.5
BuGal C16:1	9.9	14.0	1.8	12.7	19.0	28.8	2.2
BuGal C18:2	9.4	13.3	1.5	12.0	18.0	29.8	1.2
BuGal C18	9.4	15.2	1.6	12.6	19.8	28.0	3.0
BuGal C18:1	9.4	14.2	1.5	12.3	18.9	28.9	2.1
Microbial SFAE	9.6	15.3	2.1	12.7	19.9	27.9	3.1

**Fig. 4:**
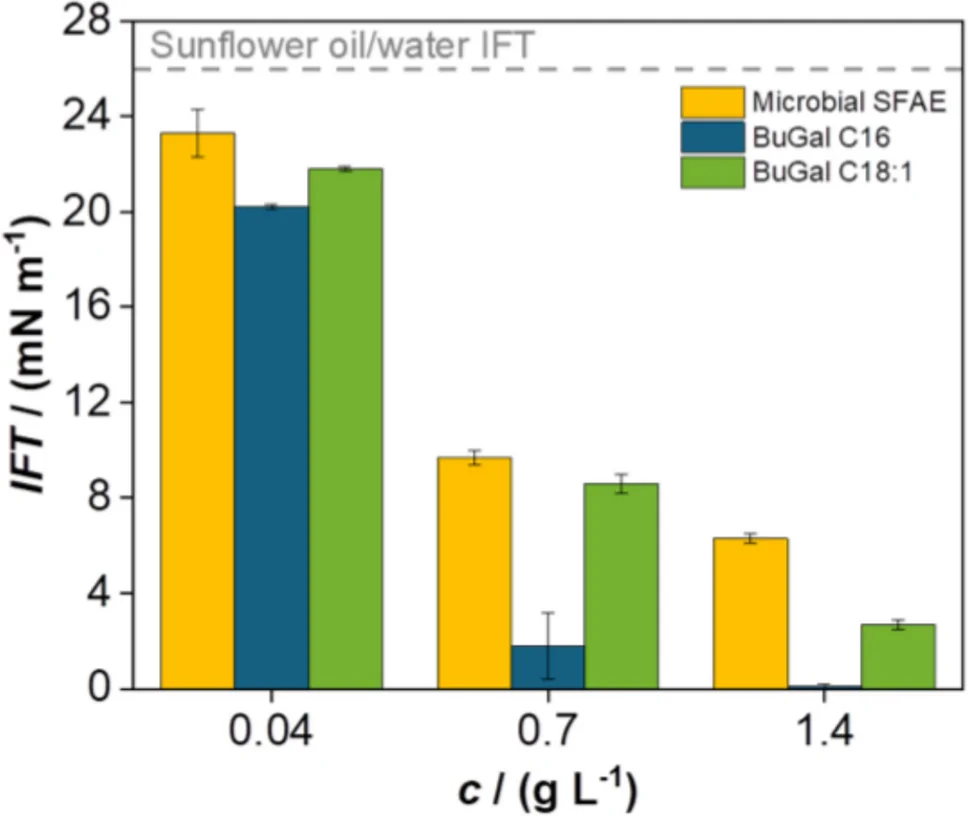
Sunflower oil/water interfacial tension (IFT) values at increasing concentrations (0.04, 0.7, and 0.14 g L^−1^) of the microbial SFAE, *n*-butyl 6-*O*-palmitoyl-β-d-galactopyranoside (reported in Semproli et al. 2023) and *n*-butyl 6-*O*-oleoyl-β-d-galactopyranoside

To evaluate the potential of the produced bio-based surfactants via such a biotechnological process, the interfacial tension (IFT) reduction of the microbial SFAE mixture at sunflower oil/water (Milli-Q) interface was assessed. As shown in Fig. 4, the synthesized mixture reduced the IFT from 26 mN m⁻^1^ (the baseline value without surfactant) to 6.3 mN m⁻^1^ at a concentration of 1.4 g L⁻^1^. The microbial SFAE mixture consists of approximately 50% saturated fatty acid tails—primarily palmitic acid (C16, 45%)—and 50% unsaturated tails, with oleic acid (C18:1) accounting for about 40% of the composition. It is well established that the interfacial behaviour of a surfactant is strongly influenced by both the fatty acid chain length and its degree of unsaturation (Allen and Tao 2002; Sun et al. 2009; Wang et al. 2021). To better understand the impact of unsaturated chains on interfacial properties, *n*-butyl 6-*O*-oleoyl-β-d-galactopyranoside (BuGal C18:1) was synthesized and characterized. The IFT results for the microbial mixture and BuGal C18:1 were compared with previously reported data for *n*-butyl 6-*O*-palmitoyl-β-d-galactopyranoside (BuGal C16) (Semproli et al. 2023). At the same concentration (1.4 g L⁻^1^), BuGal C16 and BuGal C18:1 achieved significantly lower IFT values—0.1 and 2.7 mN m⁻^1^, respectively—compared to the microbial mixture (6.3 mN m⁻^1^), indicating superior interfacial activity of the single-component surfactants. Among them, BuGal C16 showed the most effective IFT reduction (Fig. 4).

## Discussion

Surfactants are widely used in everyday life, from personal care products and detergents to foods and medicines (Hayes and Smith 2019). The market size for these compounds was estimated at 18.85 million tons in 2025, and it is projected to reach 22.18 million tons by 2030 (Mordor Intelligence 2025). Currently, most surfactants are produced from non-renewable, petroleum-based sources. These surfactants often have poor biodegradability, leading to their accumulation and persistence in aquatic environments where they can negatively impact the microbial populations, fish, and other aquatic life (Nagtode et al. 2023; Ng et al. 2023). With over 15 million tons of surfactants used globally each year (60% of which ends up in the water systems) and the upcoming depletion of oil reserves, replacing these petroleum-based compounds with sustainable, biomass-based alternatives is an urgent need (Nagtode et al. 2023; Ng et al. 2023).

Recently, WP was used to produce the polar head of sugar-based surfactants. Specifically, lactose contained in WP was first hydrolyzed into its monosaccharide components (i.e., Glc and Gal) by a soluble β-galactosidase. Following freeze-drying, this sugar mixture was subsequently reacted in a 1-BuOH/H_2_O system using various acid resins as catalysts (Fischer glycosylation), resulting in a complex mixture of eight alkyl glycoside isomers that differ for the type of sugar (Glc or Gal), the size of the glycoside ring (pyranosides or furanosides), and the configuration of the anomeric position (α or β). This polar head mixture was subsequently esterified with commercial PA as the apolar tail, obtaining the corresponding SFAE. According to the assessment of their interfacial properties, six-membered pyranoside SFAE were significantly more effective at reducing interfacial tension at lower concentrations compared to those with a five-membered furanoside ring (Ballabio et al. 2024). Previous studies using pure glucose- and galactose-based polar heads demonstrated that SFAE with a galactose-based polar head were more effective at reducing interfacial tension than those with a glucose-based polar head (Sangiorgio et al. 2022; Semproli et al. 2023). Furthermore, among the galactose-based SFAE, the pure *n*-butyl 6-*O*-palmitoyl-β-d-galactopyranoside proved to be the most effective in creating stable emulsions, highlighting its superior interfacial properties (Semproli et al. 2023).

In this work, the pure *n*-butyl β-d-galactopyranoside derivative was synthesized directly from WP by enzymatic transglycosylation without any dilution or pre-treatment of the biomass (i.e., hydrolysis and freeze-drying).

On the other hand, microbial lipids, obtained through the fermentation of WP, were a mixture of triglycerides with C16 and C18 fatty acid chains having a varying degree of unsaturation. Fermentation-based microbial lipids can be a sustainable and cost-effective alternative to traditional oilseeds. Microbes have a shorter life cycle compared to plants, which results in a faster production cycle and higher yields. Additionally, their production through the fermentation process is not dependent on specific climates or locations; it reduces the need for extensive land use and allows for a wide range of waste streams to be used as a carbon source (Tomás-Pejó et al. 2021). Once produced, microbial lipids have to be extracted and purified from other cellular components. This recovery process typically requires large amounts of solvents and a high energy demand. Additionally, in order to be used as apolar tails for the synthesis of SFAE, the extracted lipids should be further processed into free fatty acids or alkyl esters (Gufrana et al. 2022). To overcome these issues, in this work a one-step in situ transesterification approach was set up. By treating freeze-dried cells directly with EtOH in the presence of an acid-catalyst, this method allowed the simultaneous extraction and derivatization of the intracellular lipids into ethyl esters. This one-step process significantly streamlines production, reducing both solvent usage and energy consumption.

The fully bio-based building blocks obtained from WP were coupled together by enzymatic transesterification, giving a mixture of *n*-butyl 6-*O*-acyl galactopyranosides with an overall yield of 16% (considering the two-step enzymatic process: transglycosylation and transesterification reactions). To the best of our knowledge, few papers have described such an integrated process for the preparation of surfactants starting from a single renewable biomass that is used to produce both the polar head and the apolar tail of the surfactant. Beechwood was treated with a cellulase cocktail in order to hydrolyze the cellulose fraction into simple sugars (i.e., glucose and xylose). The cellulose-hydrolyzed fraction was incorporated in a deep eutectic solvent (DES) with choline chloride and was used as the polar head of the surfactant. The same biomass was also used as a carbon source for the fermentation of the oleaginous yeast *Cryptococcus curvatus*, which accumulated fatty acids (hydrophobic tails) that were then converted into the corresponding methyl esters (FAME). SFAE were obtained by enzymatic transesterification of the sugar DES with FAME. Only a small amount of the purified product was obtained for analytical characterization (Siebenhaller et al., 2018).

Peach palm fruit shells represent a further example of a single biomass that was used as the source for both building blocks of the surfactant. Fatty acids were extracted with EtOH, and the de-oiled biomass was submitted to three sequential enzymatic reactions to recover simple sugars. The highest conversions of surfactants were in the range 19–25% (Hoyos et al. 2021). While these approaches are both compelling “all in one” processes, the isolated yield of the resulting SFAE was not reported.

Compared to previous synthesized SFAE that contained only a single apolar tail (i.e., PA) (Sangiorgio et al. 2022, 2024; Semproli et al. 2023; Ballabio et al. 2024), microbial SFAE are a mixture of different fatty acid tails with varying degrees of unsaturation (one or two unsaturations due to the presence of palmitoleic/oleic and linoleic acid, respectively). To understand how this mixture of apolar tails affects the physico-chemical properties of SFAE, its ability to reduce the interfacial tension was studied. In this case, the microbial mixture was less effective at reducing interfacial tension compared to the single-component surfactants with either palmitic or oleic acid as apolar tails. Palmitic (saturated) and oleic (monounsaturated) tails in surfactants strongly influence the interfacial tension primarily due to their packing efficiency and structural rigidity. Palmitic acid (C16), with its straight, saturated hydrocarbon chain, enables surfactant molecules to pack tightly and uniformly at the oil–water interface. This results in denser molecular coverage and more rapid saturation of the interface, thereby lowering interfacial tension more effectively (Lu et al. 2024). In contrast, oleic acid (C18:1) contains a *cis*-double bond that introduces a kink in the hydrocarbon tail, disrupting tight packing and reducing overall molecular ordering. As a result, oleic-containing surfactants form looser, less organized interfacial films, which are less effective at lowering interfacial free energy (Lu et al. 2024). Additionally, saturated chains like palmitic acid can align parallel to one another, forming a compact and cohesive hydrophobic layer. The kinked structure of oleic acid, however, limits such alignment, decreasing packing density and weakening the ability of the surfactant to reduce interfacial tension (Bhadani et al. 2017). Moreover, in the microbial-derived SFAE containing a mixture of palmitic (45%) and oleic (40%) tails, the presence of both saturated and unsaturated chains can lead to structural mismatches at the interface. This heterogeneity impairs uniform packing and may result in less efficient coverage of the oil–water interface. Saturated palmitic tails tend to form rigid, crystalline domains, while the inclusion of oleic tails can induce microphase separation or domain irregularities. Such structural disruption reduces film stability and increases interfacial tension compared to systems with homogeneous tail composition. However, despite the slightly lower interfacial performance of the microbial-derived SFAE mixture compared to the single-component surfactants, the results remain highly promising. This study complements existing literature on this topic, which has already explored the impact of different sugar moieties (Glc or Gal, pure or mixed), anomeric configurations (α/β, pure or mixed), ring size (furanosides or pyranosides, pure or mixed), and various pure fatty acid chains (C12, C16, C18) on SFAE properties.

In conclusion, this work outlines how an integrated biotechnological process can result in dairy waste valorization by yielding valuable bioproducts.

## Supplementary Information

Below is the link to the electronic supplementary material.

ESM1 (DOCX 4.27 MB)

## Data Availability

Data and material for this article are available upon request.
