# Supplementary material for: Upcycling cheese whey permeate into fully bio-based surfactants through fermentation and biocatalysis
Source: Appl Microbiol Biotechnol. 2025 Dec 18;109:270. doi: 10.1007/s00253-025-13630-y (PMC12718223; doi:10.1007/s00253-025-13630-y)
Supplement: Supplementary file 1 [file 253_2025_13630_MOESM1_ESM.docx]

**Supplementary materials for**

**Upcycling cheese whey permeate into fully bio-based surfactants through fermentation and biocatalysis**

Riccardo Semproli^1^, Lorenza Cassano^1^, Giorgia Ballabio^2^, Giuseppe Cappelletti^2^, Giovanna Speranza^2^, Silvia Donzella^3^, Concetta Compagno^3^, Daniela Ubiali^1^ and Marina Simona Robescu^1*^

^1^Department of Drug Sciences, University of Pavia, Viale Taramelli 12, I-27100, Pavia (Italy)

^2^Department of Chemistry, University of Milan, via Golgi 19, I-20133, Milan (Italy)

^3^Department of Food, Nutrition and Environmental Sciences, University of Milan, via Mangiagalli 25, I-20133, Milan (Italy)

Corresponding author e-mail address: marinasimona.robescu@unipv.it (M.S.R.)

**Table of Contents**

Supplementary Figures

**Fig. 1** ^1^H-NMR spectrum of 1-butyl β-d-galactopyranoside obtained from WP biotransformation (DMSO-*_d6_*).…..	1

**Fig. 2** GC-MS analysis of microbial FAEE obtained after one-pot extraction and derivatization from *C. oleaginous* freeze-dried cells (A) and of FAME obtained by alkaline-catalysis of microbial SFAE mixture (B)………………	2

**Fig. 3** ^1^H-NMR (A) and ^13^C-NMR (B) spectra of the microbial SFAE mixture (DMSO-*_d6_*)*.*……………………...	3

**Fig. 4** ^1^H-NMR spectrum of *n*-butyl 6-*O*-oleoyl-β-d-galactopyranoside (DMSO-*_d6_*)...............................................	4

**Fig. 5** HPLC-ELSD monitoring of WP transglycosylation reaction……………………………………………..….	5

**Fig. 6** Sample of solvent-free esterification of BuGal with PA at 80 °C……………………………………………	6

**Fig. 1** ^1^H-NMR spectrum of 1-butyl β-d-galactopyranoside obtained from WP biotransformation (DMSO-*_d6_*).

**Fig. 2** **A**) GC-MS analysis of microbial FAEE obtained after one-pot extraction and derivatization from *C. oleaginous* freeze-dried cells. **B**) GC-MS analysis of FAME obtained by alkaline-catalysis of microbial SFAE mixture.

C16:1= palmitoleyl derivative; C16:0= palmitoyl derivative; C18:2= linoleyl derivative; C18:0= oleyl derivative; C18:0= stearyl derivative.

**Fig. 3** ^1^H-NMR (**A**) and ^13^C-NMR (**B**) spectra of the microbial SFAE mixture (DMSO-*_d6_*).

**Fig. 4** ^1^H-NMR spectrum of *n*-butyl 6-*O*-oleoyl-β-d-galactopyranoside (DMSO-*_d6_*).

**Fig. 5** HPLC-ELSD monitoring of WP transglycosylation reaction: A) t_0_ (light grey), t_0.5h_ (grey), t_2.5h_ (black); B) t_2.5h_ (black) and t_24h_ (grey).

**Fig. 6** Sample of solvent-free esterification of BuGal with PA at 80 °C (endpoint: 4 h). From left to right: CalB, TLL, and SFAE (standard). Eluent: EtOAc/Hex (80:20); detection by Ce(SO_4_)_2_/(NH_4_)_6_Mo_7_O_24_x4H_2_O) in 6% v/v sulfuric acid in H_2_O and heating.
